# Development of a versatile electrochemical cell for *in situ* grazing-incidence X-ray diffraction during non-aqueous electrochemical nitro­gen reduction

**DOI:** 10.1107/S1600577523006331

**Published:** 2023-08-18

**Authors:** Sarah J. Blair, Adam C. Nielander, Kevin H. Stone, Melissa E. Kreider, Valerie A. Niemann, Peter Benedek, Eric J. McShane, Alessandro Gallo, Thomas F. Jaramillo

**Affiliations:** a Chemical Engineering, Stanford University, 443 Via Ortega, Stanford, CA 94305, USA; b SUNCAT Center for Interface Science and Catalysis, SLAC National Accelerator Laboratory, 2575 Sand Hill Rd, Menlo Park, CA 94025, USA; c Stanford Synchrotron Radiation Lightsource, SLAC National Accelerator Laboratory, 2575 Sand Hill Rd, Menlo Park, CA 94025, USA; d Research Department, Sila Nanotechnologies, 2470 Mariner Square Loop, Alameda, CA, USA; Advanced Photon Source, USA

**Keywords:** non-aqueous Li-mediated electrochemical nitrogen reduction, synchrotron X-ray diffraction, electrocatalysis, electrochemical cell design, grazing incidence, *in situ*, solid electrolyte interphase

## Abstract

*In situ* techniques are essential to understanding the behavior of electrocatalysts under operating conditions. When employed, *in situ* synchrotron grazing-incidence X-ray diffraction (GI-XRD) can provide time-resolved structural information of materials formed at the electrode surface. *In situ* cells, however, often require ep­oxy resins to secure electrodes, do not enable electrolyte flow, or exhibit limited chemical compatibility, hindering the study of non-aqueous electrochemical systems. Here, a versatile electrochemical cell for air-free *in situ* synchrotron GI-XRD during non-aqueous Li-mediated electrochemical N_2_ reduction (Li-N_2_R) has been designed. This cell not only fulfills the stringent material requirements necessary to study this system but is also readily extendable to other electrochemical systems. Under conditions relevant to non-aqueous Li-N_2_R, the formation of Li metal, LiOH and Li_2_O as well as a peak consistent with the α-phase of Li_3_N was observed, thus demonstrating the functionality of this cell toward developing a mechanistic understanding of complicated electrochemical systems.

## Abstract 2

A new electrochemical cell design enables air-free *in situ* synchrotron grazing-incidence X-ray diffraction measurements of Li-containing species during non-aqueous Li-mediated electrochemical nitro­gen reduction. Such measurements will facilitate the development of a mechanistic understanding of the electrode–electrolyte interface under Li-N_2_R conditions, allowing for the rational design of Li-N_2_R toward improved Faradaic efficiency toward NH_3_.

## 1. Introduction

The electrochemical production of fuels and other products is receiving significant attention as the world transitions away from fossil-fuel-driven processes. Among the reactions of interest, focus has been placed on the electrochemical reduction of N_2_ to NH_3_ (Giddey *et al.*, 2017 ▸; Westhead *et al.*, 2021 ▸), which, when coupled to renewable electricity, promises an environmentally sustainable alternative to the energy-intensive CO_2_-emitting industrial Haber–Bosch process (Erisman *et al.*, 2008 ▸; MacFarlane *et al.*, 2020 ▸). Non-aqueous Li-mediated electrochemical N_2_ reduction (Li-N_2_R) has been identified as a promising route toward sustainable point-of-use NH_3_ synthesis, and gaining a mechanistic understanding of this reaction will be invaluable in developing a system that operates at commercially relevant conditions and product yields. Understanding how the formation of intermediates and other species involved in Li-N_2_R relates to system parameters and descriptors (*e.g.* proton source, Li salt identity, applied potential, organic solvent, Faradaic efficiency toward NH_3_) would provide insight into the mechanisms by which these components interact to convert Li_3_N to NH_3_. However, the air reactivity of Li-based system components complicates such measurements as it renders *ex situ* characterization of the electrode surface unrepresentative of the electrode surface, even immediately after a reaction (Tsuneto *et al.*, 1994 ▸; Lazouski *et al.*, 2019 ▸; Suryanto *et al.*, 2019 ▸).

*In situ* methods are thus required to obtain information related to catalyst structure and electronic state under electrochemical reaction conditions. However, such measurements are often challenging, with each characterization technique often requiring specific electrochemical cell and sample geometries (Bak *et al.*, 2018 ▸; Farmand *et al.*, 2019 ▸; Sottmann *et al.*, 2019 ▸; Liu *et al.*, 2016 ▸). Furthermore, electrochemical cells designed for such purposes often suffer from a lack of reusability or incompatibility with certain system materials (Farmand *et al.*, 2019 ▸). For example, 3D-printed materials are often incompatible with organic solvents, limiting the use of such materials to only a subset of aqueous electrochemical reaction systems.

In this work, we present the design of a versatile, chemically resistant, reusable electrochemical cell for *in situ* synchrotron grazing-incidence X-ray diffraction (GI-XRD) under non-aqueous Li-N_2_R conditions that is readily deployable for myriad electrochemical systems beyond Li-N_2_R, such as the aqueous electroreduction of O_2_, NO_3_^−^ and CO_2_. The cell provides flow capability and can be operated in an air-free environment at synchrotron beamlines, enabling air-sensitive experiments. Furthermore, we present GI-XRD measurements in which we demonstrate the applicability of the cell toward observation of the formation of Li-containing species under air-excluded conditions relevant to non-aqueous Li-N_2_R.

## 2. Electrochemical cell design

**Figure 1:**
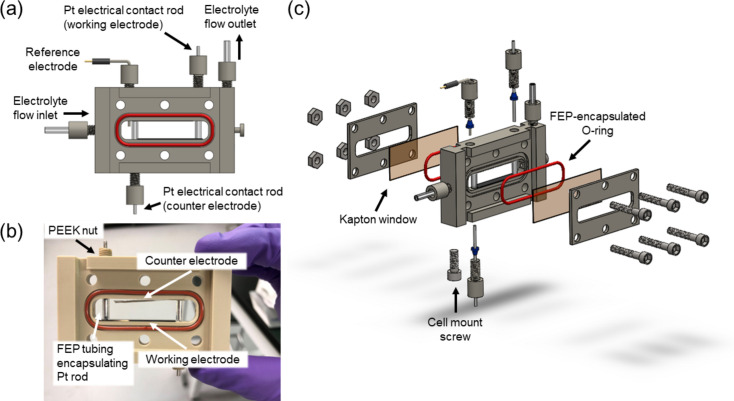
A schematic of the GI-XRD cell. The main body of the cell is presented in (*a*), with a photograph of the cell body in the opposite orientation before the cell has been fully assembled presented in (*b*). An exploded schematic of the cell parts is presented in (*c*). Although here the Pt counter electrode bends slightly toward the working electrode, giving a distance of 7.4 mm between electrodes at its closest, it would be trivial to use a thicker Pt foil to allow for a fully straight electrode.

A schematic of the electrochemical cell is presented in Fig. 1 ▸. The cell body and end plates were computer-numerical-control machined from polyether ether ketone (PEEK), which is chemically compatible with a wide range of organic solvents, including tetra­hydro­furan (THF) (da Silva Burgal *et al.*, 2015 ▸). A 35 mm × 5 mm working electrode was placed on the bottom of the chamber, while a counter electrode of the same size was placed at the top [Figs. 1 ▸(*a*) and 1 ▸(*b*)]. Both electrodes were secured using Pt rods that extended through ports in the cell body and were tightened into place using PEEK IDEX ferrules and nuts screwed into threaded ports in the cell body. The use of these rods to hold the electrodes in place and provide electrical contact to the electrodes eliminated the need for ep­oxy in the cell. Alligator clips were secured to the portion of the Pt rods extending out of the cell to make electrical contact with the potentiostat. To minimize contact of the Pt rods with the electrolyte and to avoid Li electroplating onto the Pt rod rather than the working electrode, each rod was sheathed in insulating fluorinated ethyl­ene propyl­ene (FEP) tubing cut to the length of the rod extending into the electrolyte chamber [Fig. 1 ▸(*b*)]. When FEP sheaths were not used, it was possible to visually observe the accumulation of plated material at the rod as well as the preferential deposition of material at the end of the working electrode near the Pt rod. However, the use of sheaths allowed for uniform material deposition across the entire length of the working electrode. The width of the electrolyte chamber in the direction of the X-ray beam was limited to 5 mm to minimize attenuation of the beam by the electrolyte, while still allowing for a wide enough sample to provide an easily detectable sample signal and easy sample alignment for a 17 keV incoming X-ray. The dimensions of the cell electrolyte chamber were 47 mm × 10 mm × 5 mm, while the total cell length was 37.5 mm. This resulted in an electrolyte chamber volume of 2.3 ml. A larger size in the transversal direction was employed to maximize total sample size to facilitate ammonia quantification in future studies. The cell additionally allows for experiments using either a two- or three-electrode configuration. For a three-electrode electrochemical cell configuration, a leakless PEEK Ag/AgCl reference electrode was inserted into the second threaded port in the top of the cell body and tightened into place using PEEK IDEX ferrules and nuts [Fig. 1 ▸(*a*), upper left]. This port is plugged with a PEEK IDEX plug nut for cell use in a two-electrode configuration. Finally, Kapton film windows (1 mil, 0.0254 mm) were compressed onto FEP-encapsulated silicone O-rings on either side of the main cell body between two end plates, which were secured by tightening nuts onto screws that passed through holes in the end plates and cell body [Fig. 1 ▸(*c*)].

The inlet electrolyte flow channel was positioned on the side of the electrochemical cell midway up from the bottom of the chamber [Fig. 1 ▸(*a*), left]. To allow the cell to be filled completely, ensuring contact of the counter electrode with the electrolyte, the outlet stream exits the cell body through the top of the cell [Fig. 1 ▸(*a*), right]. The assembled cell was then screwed onto a mounting plate via a threaded hole placed in the bottom corner of the cell body [Fig. 1 ▸(*c*)]. This entire cell setup was isolated in an inert environment by flowing He gas into a 3D-printed cap [Fig. S1 of the supporting information (SI)] through which inlet/outlet electrolyte tubing was passed (Cao *et al.*, 2016 ▸). This cap was required to ensure an air-free sample environment, and to facilitate air-free transport of the cell between the Ar glovebox and the beamline end station.

## 3. Validation experiments of the electrochemical cell for non-aqueous Li-N_2_R

### 3.1. Air-free setup of the electrochemical cell at a synchrotron GI-XRD beamline

**Figure 2:**
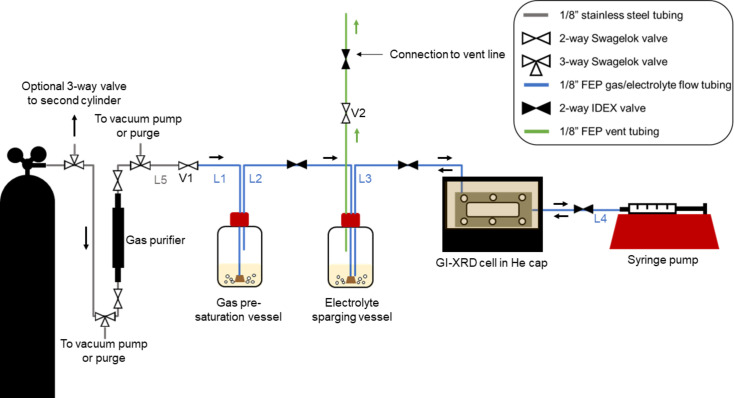
A schematic of the air-free GI-XRD setup. Electrolyte lines to the right of V1 were assembled in an Ar glovebox, where V1 and V2 were closed before transfer to the beamline. A three-way valve can be added between the cylinder and the three-way valve to the vacuum pump for ease of alternating between two gas sources.

*In situ* GI-XRD measurements in an out-of-plane geometry were performed at BL 2-1 at the Stanford Synchrotron Radiation Lightsource (SSRL) with an X-ray energy of 17 keV. The flux of the incident X-ray was ∼10^12^ photons s^−1^. The beam was relatively unfocused [spot size 150 µm (horizontal) × 50 µm (vertical)]. Thus, with this large volume of electrolyte being irradiated as the electrolyte was flowing continuously through the cell and the relatively low photon flux, minimal damage to the electrolyte was expected (Swallow *et al.*, 2022 ▸; Qiao *et al.*, 2012 ▸). Experiments carried out at open-circuit conditions did not show appreciable electrolyte damage leading to the formation of crystalline phases prior to application of a potential. It is possible that the X-ray beam results in some solvent breakdown, causing the formation of a solid electrolyte interphase (SEI) layer consisting of non-crystalline material phases. However, it is difficult to distinguish the formation of an amorphous SEI layer resulting from X-ray damage and that resulting from known degradation of THF associated with the presence of electroplated Li (Koch, 1979 ▸; Aurbach *et al.*, 1988 ▸; Zhuang *et al.*, 1998 ▸). Such a determination would require extensive further study and was consequently considered out of scope of this cell-design work. High-purity N_2_ and Ar cylinders (99.999%) were connected to a gas purifier using a three-way valve, after which the gas flowed into a gas pre-saturation vessel (Fig. 2 ▸, Figs. S2 and S3, full setup details in the SI). Pre-saturation of the gas was performed to prevent evaporation of the volatile non-aqueous solvent (THF), which would otherwise affect electrolyte component concentrations. The THF-saturated gas then flowed directly into the electrolyte (THF, 0.5 *M* LiClO_4_) in the electrolyte sparging vessel. This electrolyte was sparged continuously throughout the duration of the experiment while being pumped back and forth through the GI-XRD cell at a rate of 0.5 ml min^−1^ via a 10 ml glass syringe and syringe pump (Fig. 2 ▸, Fig. S3, see the SI for additional details). To avoid exposure of the general sample environment assembly to air, the electrochemical cell and portions of the setup presented in Fig. 2 ▸ were assembled in an Ar glovebox before being brought to the beamline (see the SI), such that setup components were always in an inert environment. Once at the beamline end station, the cap encasing the cell was attached to a He line and purged continuously (Fig. S1).

### 3.2. GI-XRD measurements during chronopotentiometry for observation of Li-containing species

**Figure 3:**
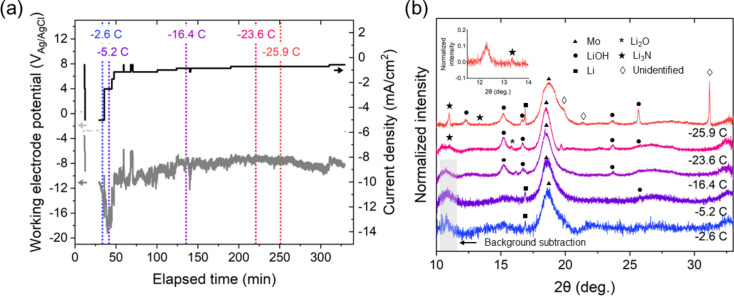
Electrochemical behavior over time of the thin-film Mo cathode in the electrochemical cell is presented in (*a*). The electrolyte consisted of N_2_-saturated THF with 0.5 *M* LiClO_4_. Current density is normalized by the geometric surface area of the working electrode (1.75 cm^2^). Potentials were measured versus a leakless PEEK Ag/AgCl reference electrode. The measured uncompensated resistance of the cell was 1233.5 Ω and was corrected using standard methods (see the SI). XRD measurements performed during the electrochemical measurements shown in (*a*) are presented in (*b*), with each diffractogram labeled with the quantity of charge passed at the time of measurement. These timepoints are indicated in corresponding colors in (*a*). Purified N_2_ was sparged into the electrolyte continuously while the electrolyte was pumped back and forth through the cell.

To confirm the suitability of this cell design for *in situ* GI-XRD measurements with the aim of observing product formation during air-free non-aqueous Li-N_2_R, we performed preliminary GI-XRD measurements under galvanostatic conditions in an electrolyte of THF, 0.5 *M* LiClO_4_, sparged with purified N_2_. The working electrode was a 50 nm Mo film deposited onto a 35 mm × 5 mm degenerately doped conductive Si wafer via physical vapor deposition (see Fig. S1), the counter electrode was a Pt foil of the same size and the reference electrode was a leakless PEEK Ag/AgCl electrode (eDAQ, ET072). The junction of this reference electrode has a resistance under 10 kΩ, and the junction potential is expected to be independent of the solvent used, according to documentation for the electrode. Because of this junction potential, we refrain from converting the reported potential to other commonly used references. Fig. 3 ▸ presents GI-XRD measurements that were executed at θ = 0.2° for various quantities of charge passed under varying applied current conditions between −5 and −0.5 mA cm^−2^ (see Fig. S4 for GI-XRD diffractograms before background subtraction). A proton source was excluded from the electrolyte for these preliminary measurements to maximize the likelihood of observing Li_3_N, which would otherwise be expected to react with the proton source to form NH_3_. Over the course of the experiment, the applied current density was decreased until a current density at which the working electrode potential was stable was reached [Fig. 3 ▸(*a*)].

Because the strongest peaks of the Li-containing species of interest are located below 2θ = 30° (Fig. S5), data were collected primarily in the 2θ range of 10–33°. *Ex situ* GI-XRD measurements of the working electrode show Mo peaks at 2θ = 18.5° and 2θ = 26.9° (Fig. S6); the peak at 2θ = 18.5° was used for normalization of all measured diffractograms. At an incident angle of 0.2°, 17 keV X-rays are expected to be nearly completely reflected from Mo, although they would be expected to entirely penetrate through a thin (∼3 nm) MoO_3_ surface layer, reducing sensitivity to the oxide (Fig. S7). While the absence of a MoO_3_ signal could be due to a lack of crystallinity of this phase, the presence of this phase in other studies using this cell (Blair *et al.*, 2023 ▸) at open-circuit conditions suggests that it is likely present in the Mo cathode samples, and therefore the lack of signal may be due to the low incident angle used. Over the course of the measurement, the Mo peak at 2θ = 18.5° was observed to shift toward higher values. Peak shifting in XRD can be associated with either compressive or tensile strain within films, with such strain introduced by a variety of factors, including voids and impurities or changes in film structure (Khatri & Marsillac, 2008 ▸). Here, the cathode thin film begins as a mixture of Mo and oxidized Mo, with the oxide being reduced over the course of the chronopotentiometry (Blair *et al.*, 2023 ▸), as well as the known tendency of Li to be intercalated into MoO_3_ (Lee *et al.*, 2008 ▸). Thus, given the dynamic nature of the surface of the thin film, there are many factors that likely introduce strain into the Mo thin film and contribute to the observed shift in the Mo peak toward larger 2θ values. As charge was passed at the cathode, multiple additional peaks that can be attributed to a variety of Li-containing species appeared. Despite the increasing roughness of the sample surface as these species were deposited onto the Mo electrode, we remained able to observe significant signal related to these compounds. Within the first −2.6 C of charge passed [Fig. 3 ▸(*b*), blue], a small sharp peak at 2θ = 16.9° corresponding to Li metal appeared [Fig. 3 ▸(*b*), Fig. S5]. The sharpness of this peak is indicative of single large crystallites of Li and is consistent with studies of Li plating in the Li-ion battery literature (Wang *et al.*, 2020 ▸; Zhao *et al.*, 2021 ▸). Between −5.2 and −23.6 C, additional peaks appear. We attribute the peaks at 2θ = 12.3, 15.2, 16.7 and 25.7° as likely belonging to LiOH, while the appearance of a peak that merges into a shoulder at 2θ = 15.2° at 2θ = 15.7–15.8° would be consistent with Li_2_O. The peaks at 2θ = 23.65, 21.3 and 31.2° are more difficult to identify, and we thus cannot confidently attribute these peaks to specific species, although it is possible that they arise from crystalline SEI components resulting from the degradation of THF due to either the presence of Li-containing species at the cathode surface or some amount of X-ray beam damage (Koch, 1979 ▸; Aurbach *et al.*, 1988 ▸; Zhuang *et al.*, 1998 ▸). However, they are not consistent with most Li-containing or possible solvent degradation species that we hypothesized could be present (Fig. S5) or the PEEK material of the cell (Fig. S8), and future work will aim to assign these peaks to chemical species.

A peak of particular interest appears at −23.6 C at 2θ = 10.98° and quickly grows in intensity by the final GI-XRD measurement at −25.9 C. This peak is close to the peak largest in intensity that would be expected for α-Li_3_N, which is located at 2θ = 10.795° at an X-ray energy of 17 keV (Fig. S5). Although this peak is slightly shifted, the presence of an additional small peak at 2θ = 13.3° in the diffractogram after −25.9 C charge was passed [Fig. 3 ▸(*b*), red, inset] further supports the assignment of Li_3_N at the Mo electrode surface. The presence of a single clear peak at the 2θ value at which the highest-intensity peak of α-Li_3_N is expected is consistent with studies of the cathode surface in Li-N_2_ batteries, which have often been unable to detect Li_3_N peaks at higher 2θ values (Ma *et al.*, 2017 ▸; Markowitz & Boryta, 1962 ▸).

This apparent Li_3_N peak appears only after a significant quantity of charge has been passed. This suggests that the formation of Li_3_N in this system is either slow, which could be related to the low solubility of N_2_ in THF limiting the quantity of N_2_ that reaches the plated Li to form Li_3_N, or that the layer of crystalline Li_3_N present at the cathode surface is too thin to be detected via GI-XRD at smaller amounts of charge passed. These preliminary measurements consequently suggest that a mixture of LiOH, Li_2_O and Li forms readily at the cathode surface upon application of a negative current density under these non-aqueous conditions, but it takes a much longer time for a species consistent with Li_3_N to accumulate and crystallize such that it is observable via GI-XRD.

Our preliminary results demonstrate that this cell enables observations related to the formation of Li_3_N under conditions relevant to non-aqueous Li-N_2_R. Given the demonstrated viability of this cell under such stringent conditions, it could readily be adapted to the study of catalyst materials in electrochemical systems that are less air and water sensitive, such as aqueous O_2_ reduction and evolution systems, as well as CO_2_ and NO_3_^−^ reduction systems.

## 4. Conclusions

We have presented the design of an electrochemical cell for air-free *in situ* GI-XRD measurements under non-aqueous Li-N_2_R conditions with the added features of electrolyte flow capability and chemical compatibility without requiring the use of ep­oxy or other adhesives. The functionality of the cell for *in situ* non-aqueous Li-N_2_R experiments revealed that several Li-containing species formed during the application of a N_2_R-relevant current density to a cathode consisting of a Mo film deposited onto a Si wafer. More specifically, we observed Li metal along with oxidized forms of Li such as LiOH and Li_2_O. Additionally, we measured the formation of a peak consistent with the α-phase of Li_3_N. We have demonstrated the applicability of this electrochemical cell in working toward gaining a mechanistic understanding of species at the electrode–electrolyte interface in a complex electrochemical system. This provides important foundations for further *in situ* synchrotron and electrochemical studies.

## 5. Related literature

The following references, not cited in the main body of the paper, have been cited in the supporting information: Giants (1994 ▸); Landers *et al.* (2021 ▸); Toney & Brennan (1989 ▸).

## Supplementary Material

Supporting information. DOI: 10.1107/S1600577523006331/vy5012sup1.pdf
