# Supplementary material for: Development of a versatile electrochemical cell for in situ grazing-incidence X-ray diffraction during non-aqueous electrochemical nitro­gen reduction
Source: J Synchrotron Radiat. 2023 Aug 18;30(Pt 5):917–22. doi: 10.1107/S1600577523006331 (PMC10481268; doi:10.1107/S1600577523006331)
Supplement: Supplementary file 1 [file s-30-00917-sup1.pdf]

JOURNAL OF SYNCHROTRON RADIATION

Volume 30 (2023)

Supporting information for article:

Development of a versatile electrochemical cell for *in situ* grazing-incidence X-ray diffraction during non-aqueous electrochemical nitrogen reduction

Sarah J. Blair, Adam C. Nielander, Kevin H. Stone, Melissa E. Kreider, Valerie Niemann, Peter Benedek, Eric J. McShane, Alessandro Gallo and Thomas F. Jaramillo

S1. Sample preparation for Li-N_2R

A custom-built electron-beam physical vapor deposition system (Technical Engineering Services) was used to deposit thin films of Mo onto 35 x 5 mm Si wafer substrates. The metal source target (Mo, 99.95% purity) was used as received from Kurt J. Lesker. Prior to deposition, the 500-µm-thick Si wafer substrates (SEMI Standards, <100>, p-type, boron-doped, 0.1-0.9 Ω-cm) were sonicated for 30 minutes first in soapy water, followed by 30 minutes in a 5:4:1 mixture of deionized water (18 MΩ cm): isopropanol: acetone and 30 minutes in deionized water (18 MΩ cm). 50 nm of Mo were then deposited onto the substrates at a rate of 0.1 Å /s.

S2. Electrolyte preparation for Li-N_2R

Tetrahydrofuran (THF) (Sigma-Aldrich, Inc., anhydrous, inhibitor-free, 99.9%) was dried overnight over molecular sieves (Sigma-Aldrich, Type 3A, bead size 4-8 mesh) that were activated by heating to 200 °C under Ar for 15 hours and then dried under vacuum in a glovebox antechamber. $LiClO_4$ (battery-grade, dry, 99.99% trace metals basis, Sigma-Aldrich, Inc.) was opened and stored in the Ar glovebox. The electrolyte (THF, 0.5 M $LiClO_4$) was mixed in the Ar glovebox immediately before use.

S3. Cell assembly for Li-N_2R

The PEEK cell was first dried in an oven at a temperature just above 100 °C for several hours, after which the cell was assembled outside of the glovebox, leaving the electrolyte inlet/outlet ports open to allow for evacuation of the cell in the glovebox antechamber. The cell was then mounted onto an Al plate attached to the base of the 3D-printed cap, and electrical wires attached to gold pins inserted through holes drilled into the cap were connected to the Pt rods (99.95%, Surepure Chemetals) and reference electrode (Innovative Instruments, LF-1.6-35-BENT) via alligator clips. The cell was subsequently brought into an Ar glovebox. Inside the glovebox, the inlet/outlet FEP tubing lines were screwed into their respective ports on the cell body, and the He cap was screwed onto the mounting base such that the cell was sealed into the cap. The He cap consisted of a 3D-printed cap with Kapton windows glued to the material such that the cap was transparent to incoming and outgoing X-ray beams.

30 mL of dry THF was placed into a 100-mL Pyrex vessel with an HPLC cap assembly (Chemglass Life Sciences, PTFE bulkhead) for saturation of the gas used to sparge the electrolyte with THF. 20 mL of electrolyte was placed into a second 100-mL Pyrex sparging vessel also equipped with an HPLC cap assembly. A pre-saturation vessel was utilized to saturate the gas used to sparge the electrolyte with solvent (e.g, THF). Using THF-saturated sparging gas prevented evaporation of the solvent during electrochemical experiments. As is shown in Figure S3, an inlet line (L1) was attached to the pre-saturation vessel to allow the gas to be bubbled into the THF. A closed valve (V1) was placed at the other end of this inlet line to prevent air exposure upon transport of the cell setup from the glovebox to the beam line. A second tubing line (L2) was attached to both the pre-saturation vessel and electrolyte

sparging vessel to bubble the saturated gas into the electrolyte sparging vessel. The third port in the HPLC cap assembly of the pre-saturation vessel was plugged. The outlet line from the electrochemical cell (L3) was then attached to the HPLC cap assembly of the electrolyte sparging vessel, while a vent line with a closed valve (V2) was also attached to this cap. Finally, a 10-mL glass syringe was filled with electrolyte and attached to the inlet line into the electrochemical cell (L4) via a ¼-28-to-luer-lock fitting. With the electrochemical cell positioned vertically such that the inlet line was located at the bottom of the setup, the cell was filled using the syringe until L3 was full of electrolyte (i.e. until no bubbles were seen at the outlet of L3 in the electrolyte sparging vessel. This typically required ~8 mL of electrolyte, leaving 2 mL in the syringe) to avoid pumping Ar bubbles through the cell. All electrolyte flow tubing was chemically-resistant FEP tubing.

With V1 and V2 closed and all lines, including the syringe, attached, the pre-saturation vessel, electrolyte sparging vessel, electrochemical cell in the He cap, and the glass syringe were removed from the Ar glovebox and transported to the beam line (~ 2-min walk).

S4. Cell operation at the beam line

At the beam line end station, the He inlet line into the cap was attached to a He cylinder such that the cap was purged continuously with inert gas. V2 was attached to the vent line without opening the valve, and V1 was attached to L5 without opening the valve. Gases were passed from the cylinder through a NuPure gas purifier (Eliminator Model 40 PF). The portions of L5 before and after the gas purifier were separately evacuated and refilled with either N_2 (99.999% UHP, Praxair) or Ar (99.999% UHP, Praxair) three times in order to fully rid the line of air. Gas flow was then turned on and V1 opened, followed by V2. Sparging rates were adjusted by eye, with gas bubbling into each vessel via a 1/8” FEP tube. Pre-saturation of the sparging gas prevented solvent evaporation over the course of long-duration experiments. The syringe pump was programmed to pump 8 mL of electrolyte back and forth through the cell into the electrolyte sparging vessel at a rate of 0.5 mL/min before the direction of pumping was reversed. This ensured that a larger electrolyte volume was being pumped through the cell/tubing than was contained within the combination of cell + lines (~6 mL) . Future iterations of this cell setup should include either a peristaltic pump with THF-resistant tubing or a check valve to enable pumping of the electrolyte in a circular loop through the cell, allowing for fully continuous refreshment of electrolyte that is being sparged with N_2.

Wires from the potentiostat were connected to the halves of the gold pins extending outside from the cap using alligator clips in order to make electrical contact from the potentiostat to the working, counter, and reference electrodes. Electrochemistry was performed using a Biologic VMP-300 with a 2A/30V booster channel in a three-electrode configuration. Electrolyte resistance was corrected for using the “ZIR” function in the Biologic EC Lab software, which adds 85% of the measured solution resistance to the applied potential to account for ohmic losses due to the electrolyte resistance.

S5. Grazing-incidence X-ray diffraction measurements

In situ GIXRD was performed at beam line 2-1 at the Stanford Synchrotron Radiation Lightsource (SSRL) at SLAC National Accelerator Laboratory. The energy of incident X-rays was tuned to 17 keV using a LaB_6 standard reference, and measurements were performed at an incident X-ray angle of 0.2°, with a sample-to-detector distance was 707 mm. The grazing incidence angle and the Pilatus 100K area detector were controlled using a Huber two-circle goniometer, allowing for sample alignment and data collection. Data were collected using SPEC software. In general, XRD diffractograms were collected between 2θ = 9° and 2θ = 30° at 17 keV to capture the peaks of interest at low 2θ-values, with 60 points taken at 3 sec/point. This limitation in window was employed to focus on Li-containing species, which exhibit strongest peaks at 2θ-values less than 20°. The maximum exit angle was 76° for a beam exiting the cell from the middle of the sample (2.5 mm into the cell in the direction of the beam) and the cell chamber height of 10 mm. Measurements were acquired in the form of two-dimensional Pilatus images, which were then converted into 1D diffractograms by binning the pixels of the image by 2θ and normalizing by the intensity. The integrated data were background-corrected using baseline subtraction after employing a spline interpolation method to fit the baseline. Background-subtracted data were then normalized to the Mo peak at ~2θ = 18.5° for comparison between measurements. Refraction corrections were not carried out for the presented data due to the visible roughness of the material deposited onto the cathode surface.(Toney & Brennan, 1989; Landers *et al.*, 2021)

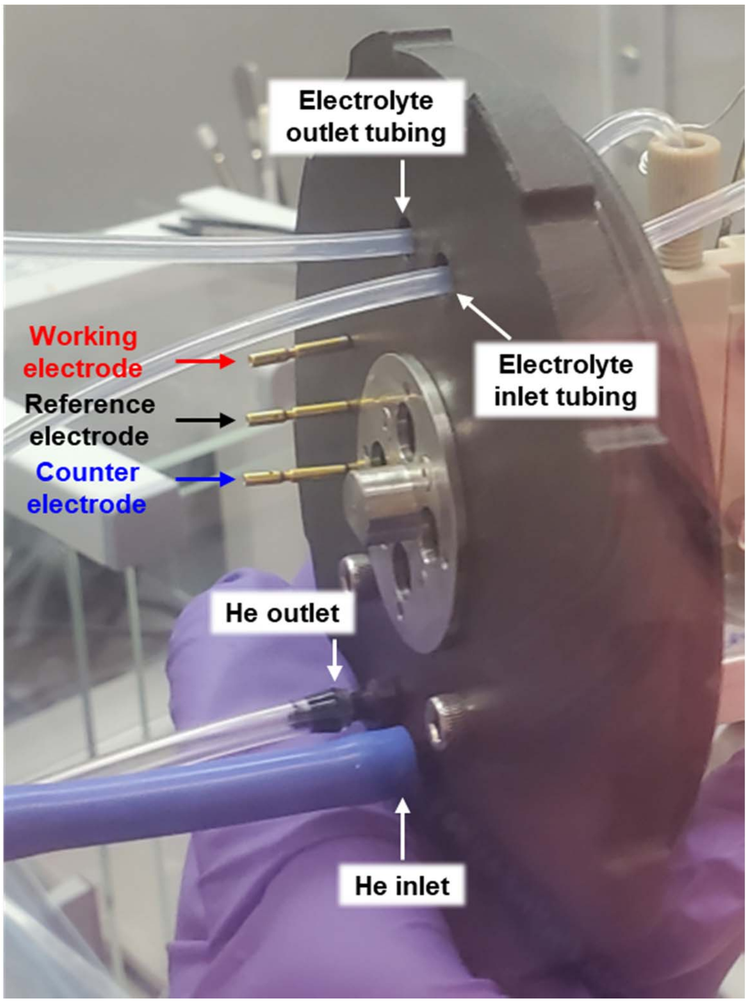

Figure S1 3D-printed base of He cap. Gold pins for electrical contact, as well as He purge inlet/outlet and electrolyte inlet/outlet tubing are shown.

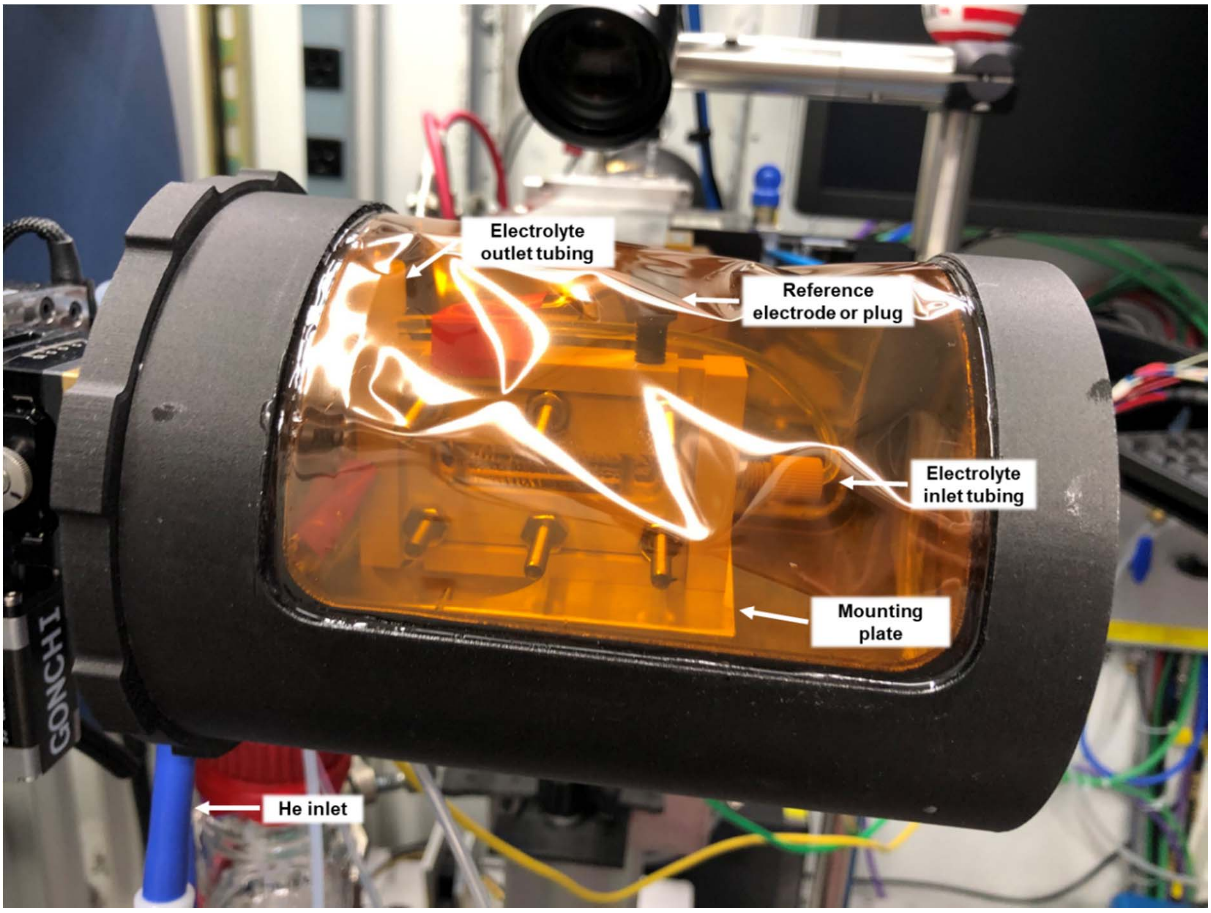

Figure S2 Electrochemical cell mounted at the beam line in the 3D-printed cap. We note that the reference electrode port can be plugged with a PEEK IDEX nut if a two-electrode measurement is desired.

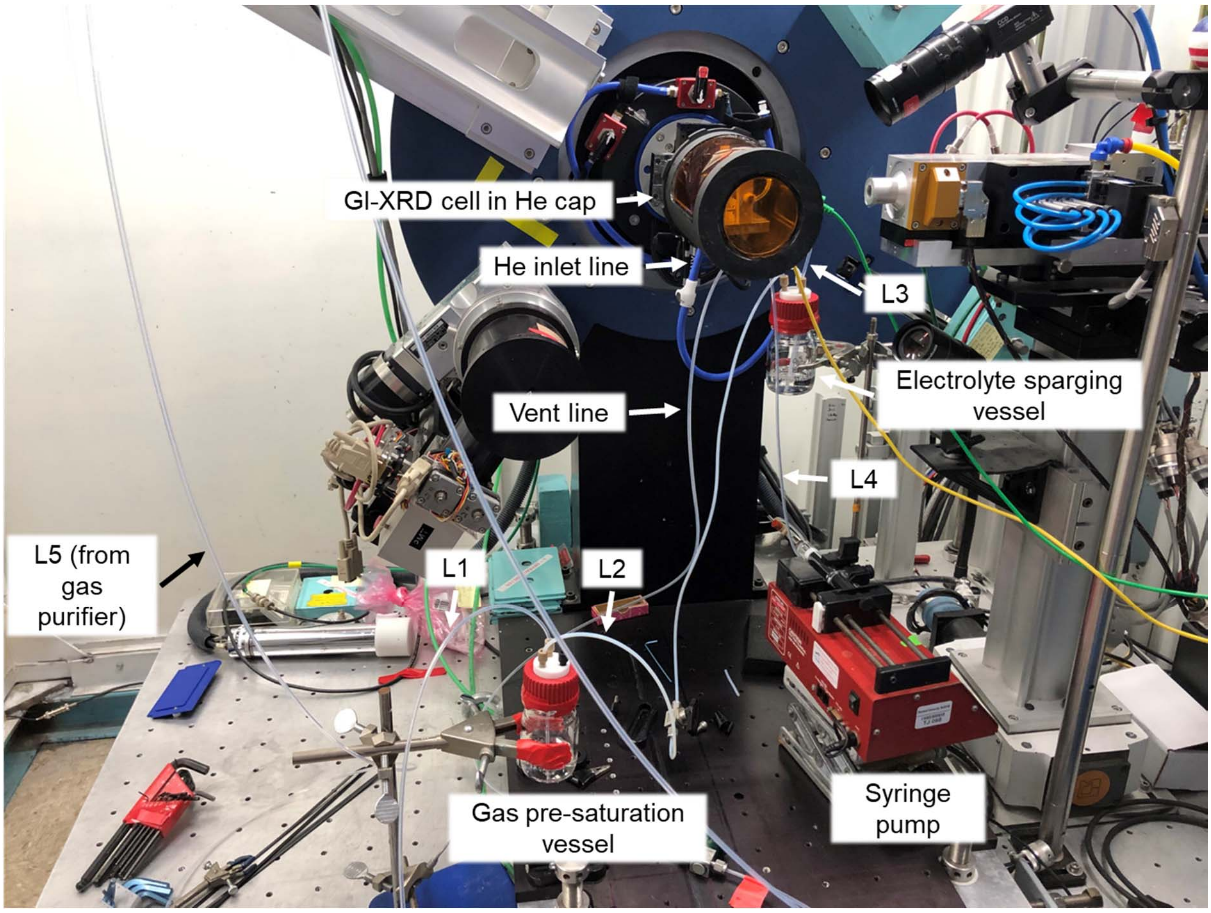

Figure S3 Electrochemical cell setup at the beam line. Labels correspond to the schematic presented in Figure 2.

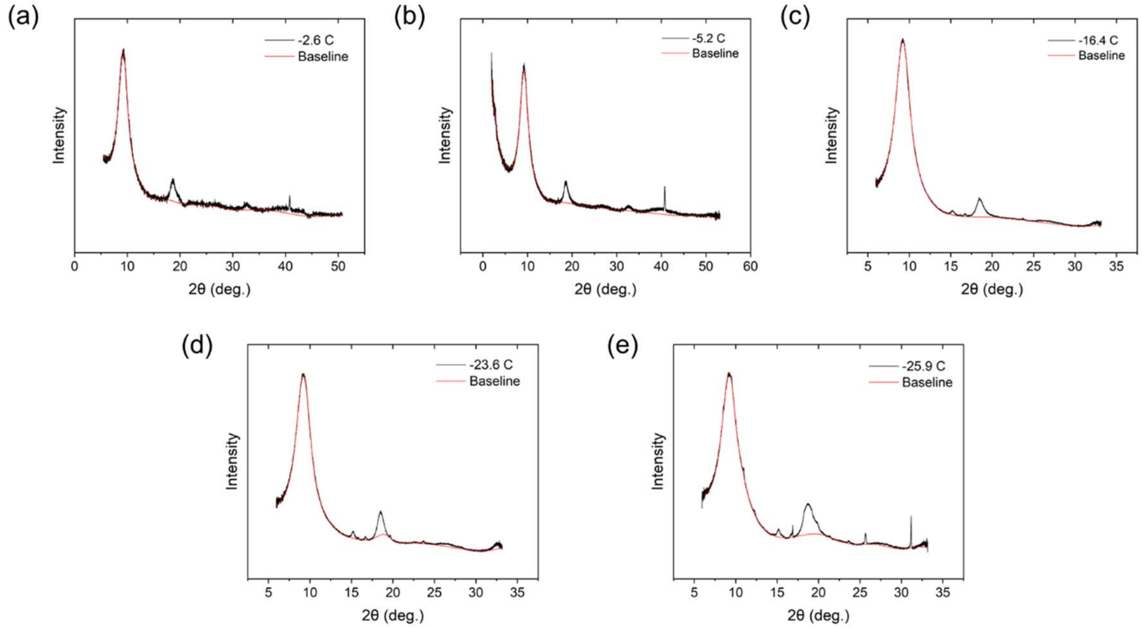

Figure S4 GI-XRD measurements corresponding to those presented in Figure 3 before background subtraction and normalization. Each panel is labeled with the charge passed at the time of XRD measurement, with baselines shown in red.

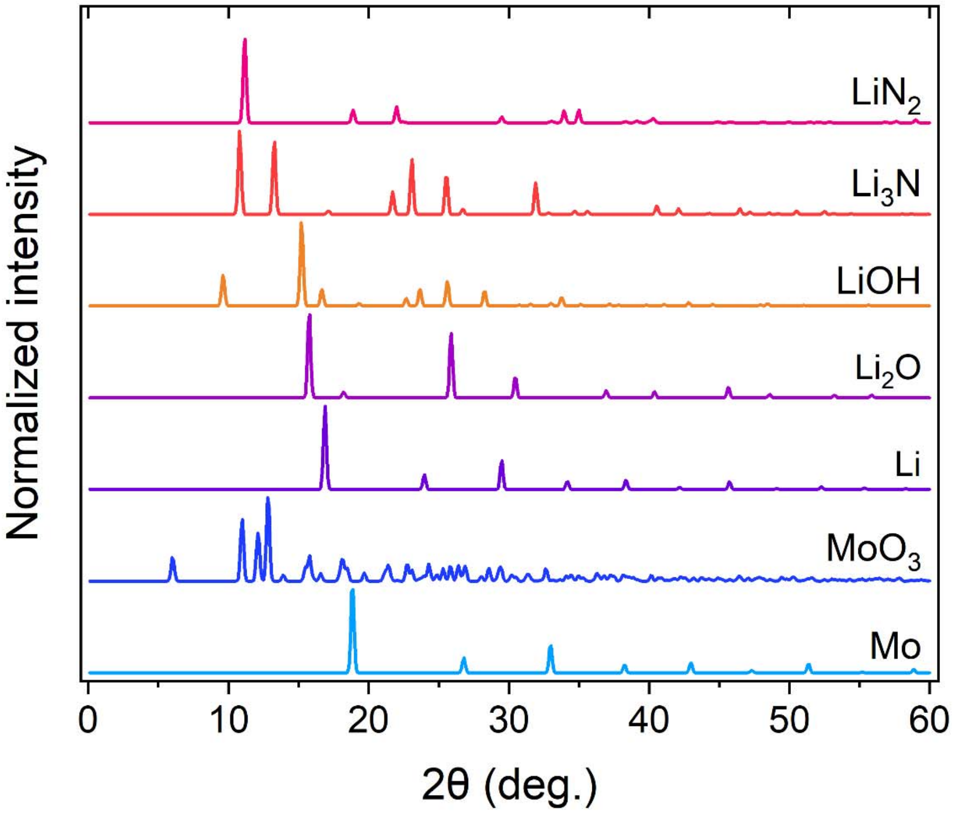

Figure S5 Reference XRD patterns from the ICSD for an X-ray energy of 17 keV. The ICSD reference patterns are Mo (52267), MoO_3 (35076), Li (44367), Li_2O (54368), LiOH (26892), Li_3N (34280), LiN_2 (25427).

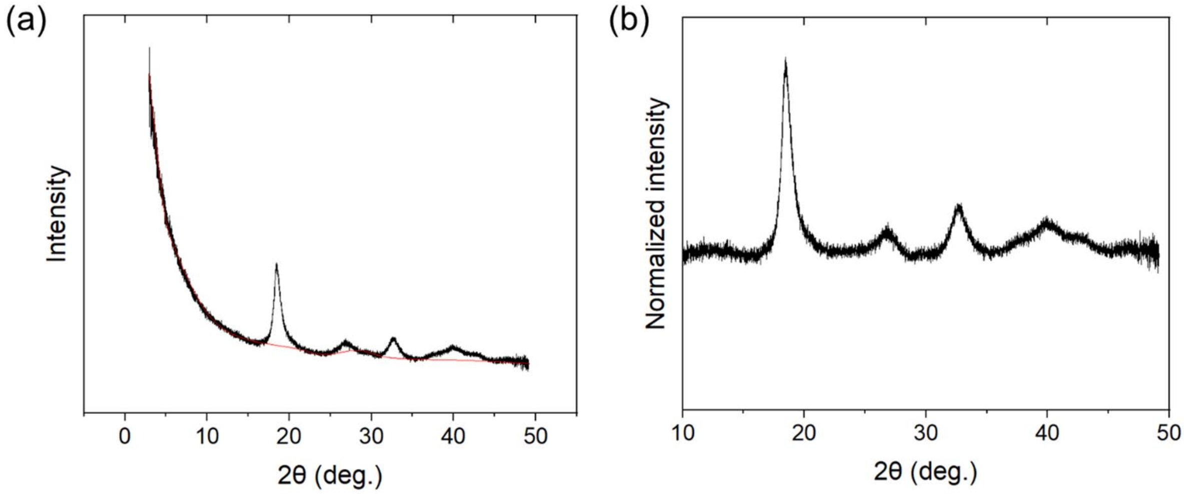

Figure S6 Ex situ GI-XRD at BL 2-1 of the sample (50 nm Mo on 35 mm x 5 mm Si wafer) mounted on the cell body without the end plates and Kapton film assembled. Raw data is shown in (a) with the background shown in red. The background-subtracted GI-XRD measurement is presented in (b), with peaks normalized to the Mo peak at 18.5°.

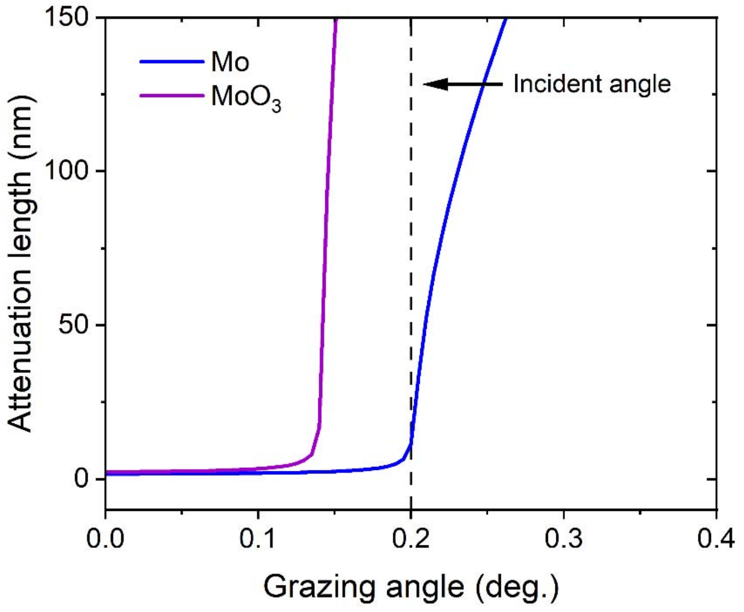

Figure S7 Calculated X-ray attenuation lengths in Mo and MoO3 for X-rays with an energy of 17 keV using the attenuation length calculator from Lawrence Berkeley National Laboratory.(X-Ray Attenuation Length) Densities of 10.217 g/cm3 (ICSD 52267) and 4.7 g/cm3 (ICSD 35076) were used for Mo and MoO3, respectively. These ICSD references correspond to those shown in Figure S5. The employed incident angle of 0.2° is shown as a dotted line for reference.

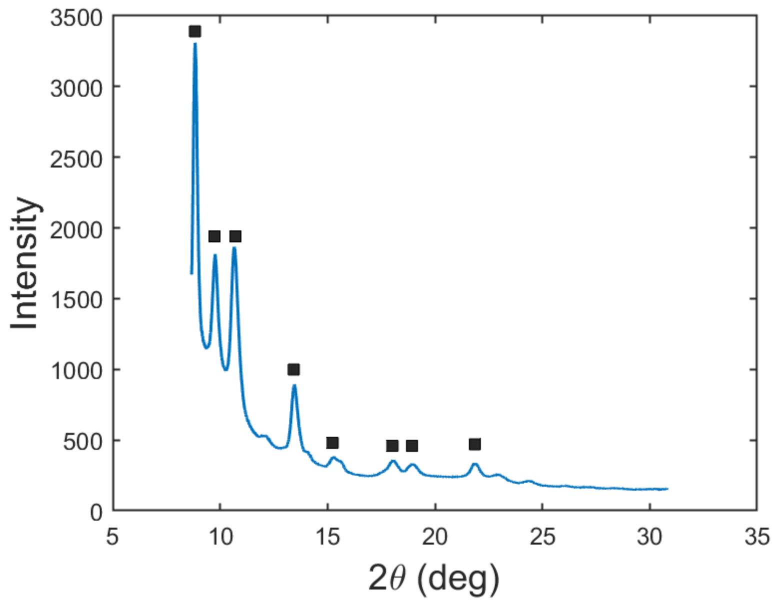

Figure S8 GI-XRD diffraction pattern with X-ray beam passing through PEEK cell material below the sample. Marked peaks are those attributed to PEEK. Peaks have not been normalized, and background has not been subtracted. (Giants, 1994)

Supporting Information References

Giants, T. (1994). *IEEE Trans. Dielectr. Electr. Insul.* **1**, 991–999.

Landers, A. T., Koshy, D. M., Lee, S. H., Drisdell, W. S., Davis, R. C., Hahn, C., Mehta, A. & Jaramillo, T. F. (2021). *J. Synchrotron Radiat.* **28**, 919–923.

Toney, M. F. & Brennan, S. (1989). *Phys. Rev. B* **39**, 7963–7966.

X-Ray Attenuation Length https://henke.lbl.gov/optical_constants/atten2.html.
